# Earth and environmental sciences ‘New Talent’ collection

**DOI:** 10.1098/rsos.251023

**Published:** 2025-07-02

**Authors:** Royal Society Open Science Editorial Team

**Affiliations:** ^1^ The Royal Society, London, England, UK

**Keywords:** environmental, sciences, talent, collections

We are pleased to introduce the Earth and environmental sciences ‘New Talent’ collection. This collection comprises invited papers which showcase some of the exciting work being funded by the Royal Society. This collection is part of a series of commissioned New Talent collections, which have already been published across a range of disciplines, including chemistry [1] and astronomy [2].

Royal Society University Research Fellowship (URF) and Newton International Fellowship (NIF) holders who are the contributors to this collection are rising stars in their areas of research. These awards are granted via a competitive grant application process to allow outstanding early-stage scientists to build their own independent research career within innovative lines of scientific study. URFs are awarded to scientists who are in their early stages of their career and have the potential to become tomorrow’s leaders in their field. Likewise, NIFs are awarded to equally promising international early career researchers looking to establish and conduct their research in the UK.

As readers will see, the collection spans the range of Earth and environmental sciences, reflecting the breadth of research areas receiving Royal Society grant support. Camejo-Harry *et al.* [3] (the first author a Royal Society NIF holder at the University of Oxford) analyse the textures and compositions of erupted material from the now extinct Grenadines archipelago within the volcanically active Eastern Caribbean. Loron [4] (a Royal Society NIF holder at the University of Edinburgh) proposes an alternative mathematical framework of decay and fossilization, relying on the change in the relative frequency and characteristics of biogenic objects within an organism–fossil system. Similarly, Anderson *et al*. [5] (the first author a Royal Society URF at the University of Oxford) discuss the evolution of eukaryotes. Within their paper, they review fossil evidence from the Proterozoic Eon (2500 to approx. 540 million years ago) and show how it can be used to understand the early evolution of eukaryotes. Zarkogiannis [6] (a Royal Society NIF at the University of Oxford) explores pelagic calcification and its effect on the surface ocean’s ability to absorb atmospheric carbon. The paper provides insights into how marine ecosystems adapt to climate change. Finally, Paluszny & Zimmerman [7] (the first author a Royal Society URF at Imperial College London) provide an overview of the role of subsurface geomechanics in the global transition towards ‘green energy’. The paper highlights the role of green energy technologies such as carbon sequestration, geothermal energy production, hydrogen storage and nuclear waste disposal.

We hope that readers of the journal will explore this collection and find something of interest. We have certainly enjoyed editing the collection, and have learnt much from those involved in this diverse and enthusiastic field. These papers are curated in this collection and are freely available at https://royalsocietypublishing.org/topic/special-collections/earth-environmental-sciences-new-talent.

Finally, we would like to thank all of the contributors to the collection and the editorial office at *Royal Society Open Science* for all their help in preparing this collection.
